# 3D Intelligent Scissors for Dental Mesh Segmentation

**DOI:** 10.1155/2020/1394231

**Published:** 2020-01-31

**Authors:** Shuai Yang, Ruikun Wang, Wenjie Zhao, Yongzhen Ke

**Affiliations:** ^1^School of Computer Science and Technology, Tiangong University, Tianjin, China; ^2^Tianjin Key Laboratory of Autonomous Intelligence Technology and Systems, Tianjin, China

## Abstract

Teeth segmentation is a crucial technologic component of the digital dentistry system. The limitations of the live-wire segmentation include two aspects: (1) computing the wire as the segmentation boundary is time-consuming and (2) a great deal of interactions for dental mesh is inevitable. For overcoming these disadvantages, 3D intelligent scissors for dental mesh segmentation based on live-wire is presented. Two tensor-based anisotropic metrics for making wire lie at valleys and ridges are defined, and a timesaving anisotropic Dijkstra is adopted. Besides, to improve with the smoothness of the path tracking back by the traditional Dijkstra, a 3D midpoint smoothing algorithm is proposed. Experiments show that the method is effective for dental mesh segmentation and the proposed tool outperforms in time complexity and interactivity.

## 1. Introduction

In the context of the emerging computer-aided medical, it is possible to acquire the 3D digital model of dental and to design and manufacture dental implant guides. Dental implant guide design systems based on digital technology have developed rapidly over the years. Typical processes of dental implants design include the following: (1) obtaining the digital model of the tooth through a traditional impression and 3D scanning and then obtaining the patient's cranial CT; (2) tooth segmentation from dental mesh, tissue segmentation, and reconstruction based on CT; (3) personalized dental implant guide design; (4) engineering analysis and manufacture; (5) implement treatment. The teeth segmentation plays a crucial role in digital dental systems (steps 2, 3, and 4). The quality of the digital dental mesh is mainly dependent on the digital dental mesh acquisition method (three-dimensional scanning technology). Therefore, it is crucial to design a user-friendly specialised tooth segmentation tool, whose quality of the segmentation is controllable.

There are three types of segmentation methods based on dental mesh: automated methods, semiautomated methods, and manual methods. Automatic methods do not require user interaction and are very convenient. However, due to the lack of controlling the quality of segmentation, such methods do not meet the accuracy requirement. Although manual methods can obtain accurate results through users' intersection, they also have many shortcomings, such as tedious and time-consuming. Semiautomated methods can keep the balance between the accuracy of segmentation and the user effort for interaction. However, the general semiautomated methods are not directly suitable for dental mesh segmentation because of the unique geometry of teeth and multiple teeth arrangements on dental mode. Moreover, existing semiautomated methods proposed to handle dental mesh have the shortcoming that the positions of interactions are not suitable for improving the segmentation accuracy.

This paper aims to develop an interactive tool for dental mesh segmentation, called 3D intelligent scissors, motivated by a user-friendly segmentation tool [1, 2]. The wire between two points inputted by user interaction is regarded as a part of the segmentation boundary. The main contribution of this work is threefold:

1. An improved intelligent scissors tool for triangle mesh segmentation has lower time complexity, which meets the requirements of real-time interaction and is specially optimised for dental mesh segmentation
2. The tool requires less interaction and can acquire better segmentation
3. An algorithm of 3D discrete curve smoothing on triangle mesh is proposed, which subtly transforms the complex problem of smoothing the curve on the surface into a simple problem of smoothing the curve on the plane

## 2. Related Work

The dental model is a triangle mesh and can be obtained by 3D scanning. Previous work about dental mesh segmentation and general mesh segmentation is reviewed. The proposed method depends on the geodesic line, and the review of the geodesic line is given at the end of the section.

### 2.1. Mesh Segmentation Approaches

There are some specific approaches [3–7] that fully exploit the dental characteristics. These methods are either interaction-intensive or result-inaccurate. Most approaches about the dental mesh segmentation reference the methods of general mesh segmentation.

Mesh segmentation is a fundamental research topic in geometry processing and computer graphics. Numerous general mesh segmentation approaches have been proposed. The manual segmentation tool is user-unfriendly, usually as a tool for obtaining benchmark segmentation [8]. Most of the mesh segmentation algorithms are automated or semiautomated and can be briefly classified as region-based and boundary-based [9].

Region-based approaches include region growing [10, 11], watershed segmentation [12, 13], and clustering [14–17]. These methods [10–17] aim to partition different regions based on similarity measures. Region-based methods regard segmentation task as finding mesh regions and grouping different regions. However, because dental meshes from patients usually have teeth crowding problems, defining a robust measure to apply region-classifying remains a challenging task.

**Figure 1:**
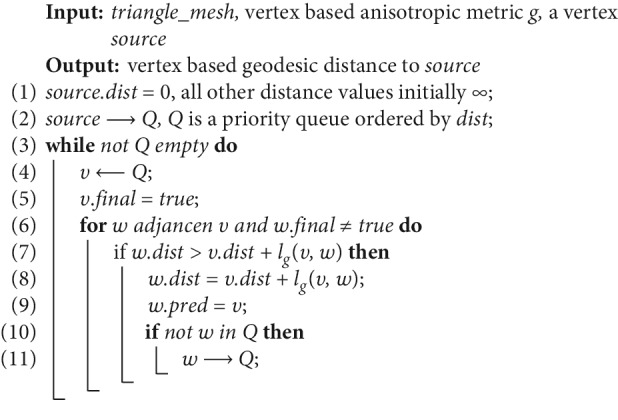
Comparison between wires obtained by our method + valley metric and Zhuang's method + Min and Max metrics.

Boundary-based methods aim to find the boundary or contour of each segmentation part. In order to select the best boundary, Golovinskiy and Funkhouser [18] proposed a method called randomized cuts. However, due to the lack of user control, the segmentation quality is poor. Zheng and Tai [19] proposed an interactive method, according to which the user interacts with the segmentation process by drawing one or more strokes across the desired boundary. Another interactive boundary-based method, called dot scissor, is discussed in [20]. With the tool, the user's effort is reduced to placing only a single click where a cut is desired. Although the interaction needed by the methods [19, 20] is little, the segmentation boundary does not fit well with the user's intentions. Zhuang et al. [1] proposed a live-wire mesh segmentation tool, with defining a wire through two endpoints entered by the user as the segmentation boundary which is the shortest path-based anisotropic metric over the surface. The method balances the ability to control results and the user effort for interaction, but the method is time-consuming, which is intolerable as it is a real-time interactive tool. In detail, the method needs mesh embedding and local subdivision at initialisation, and computing wires is time complex because it uses the exact method [21]. Moreover, the tool fails in teeth segmentation when the seeds are far apart (Figure 1).

### 2.2. Computing Geodesics Approaches

The idea of the live-wire based algorithm for mesh segmentation is to define a wire between two points on the surface as a segmentation boundary, and the critical problem is to compute the wire quickly. The geodesic-based suitable anisotropic tensor can be used as the wire, and it is a special kind of geodesic on the surface. Now, we review the work on geodesic based on triangle mesh. There are two types of methods: exact method and estimated method [21].

Chen and Han [22] proposed an exact algorithm based on the idea of one angle one split. This algorithm complexity is *O*(*n*^2^). MMP [23] algorithm is another method for computing exact geodesic-based continuous Dijkstra, and its average time complexity is *O*(*n*^1.5^ log *n*) but in the worst case is *O*(*n*^2^ log *n*). Ying et al. [24] proposed the SVG (saddle vertex graph) algorithm, which prebuilds the saddle vertex graph and can compute the shortest path through the Dijkstra algorithm. The time complexity of the algorithm is *O*(*D n* log *n*), *D* << *n*. In short, the exact method is time complex, although the SVG algorithm is better in time complexity. Moreover, all the above algorithms need to fulfil the triangle inequality everywhere, and violation of the triangle inequality is the typical behaviour especially for high degrees of anisotropy [25].

Estimated methods generally obtain the approximate solution of the discrete geodesic by solving the partial differential equation. Generally, it is faster than the exact methods. Among estimated methods, geodesic in heat [26, 27] performs very well, but it is Euler metric based. Yang and Cohen [28] extended the method by adding the variable heat transfer coefficient, and it can lead the geodesic along the feature area. Combining the anisotropic Laplace-Beltrami operators proposed by Andreux et al. [29] with the heat method, the anisotropic heat equation can be solved and then the features sensitive geodesic can be acquired. In short, all the above algorithms are faster, but they have poor robustness because solving PDE is unstable in the condition of high degrees of anisotropy.

Another estimated method is named short-term vector Dijkstra (STVD) [25]. The method uses a new version of update function by exploiting a vector-valued short-term memory that aims to improve accuracy. However, the results are unsmooth.

The conclusion of the literature review is that live-wire-based segmentation tool is user-friendly, but existing methods are time-consuming and do not exploit dental characteristics.

## 3. Our Method

Inspired by the intelligent scissors [2] in image segmentation, Zhuang et al. [1] proposed a live-wire method for mesh segmentation. In the method, the geodesic between two points on the surface is defined as a part of the segmentation boundary. However, it consumes time in computing exact geodesic and is meaningless in the segmentation application.

Similar to the live-wire segmentation, in this paper, the proposed teeth segmentation tool starts with a single click on the triangle mesh to select a seed point. Then, the program traces back a smooth path between the seed point and the current position of the mouse in real time.

**Figure 2:**
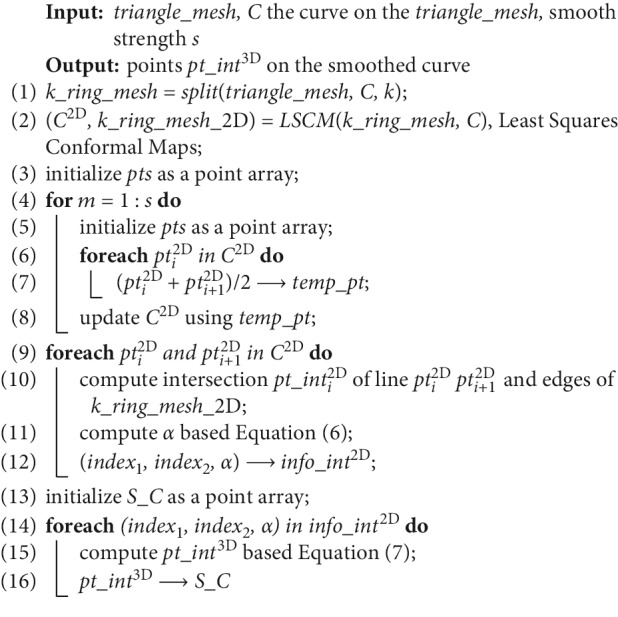
The steps of our method.

For real-time interaction, a method to fast compute the geodesic is presented. Besides, two anisotropic metrics are defined, which can force the geodesic line lying at the feature of the surface. Unfortunately, because of using an inexact method based graph search [30], the path along the edge is often unsmooth, so a 3D curve smoothing algorithm is proposed. The steps of obtaining a complete segmentation boundary are shown in Figure 2, which mainly include the following ones:

1. Initialization: computing the minimum and maximum curvature and the normal and the tensor-based anisotropic metric vertex (Section 3.1)
2. Computing the anisotropic geodesic (Section 3.2)
3. Tracking back the shortest path
4. Smoothing the path with a proposed method (Section 3.3)

### 3.1. Anisotropic Metrics

In this paper, the “shortest” path between two points (geodesic) as a part of the segmentation boundary is used. Obviously, the “shortest” is not the shortest in the usual sense (Euler metric). In order to define the concept of the “shortest”, a metric tensor *g* is introduced. In a Riemannian manifold *M*, tangent plane *T*_*a*_*M* is local Euclidean representation of manifold *M* around point *a*. Moreover, in *M*, *b* is another point around *a*. Then, the length between *a* and *b* in *M* based the metric tensor *g*_*a*_ is approximately defined as follows:

(1) $$\begin{array}{c} x=a-b,\\ d_{g}\left(a,b\right)=\sqrt{x^{T}g_{a}x}. \end{array}$$

**Figure 3:**
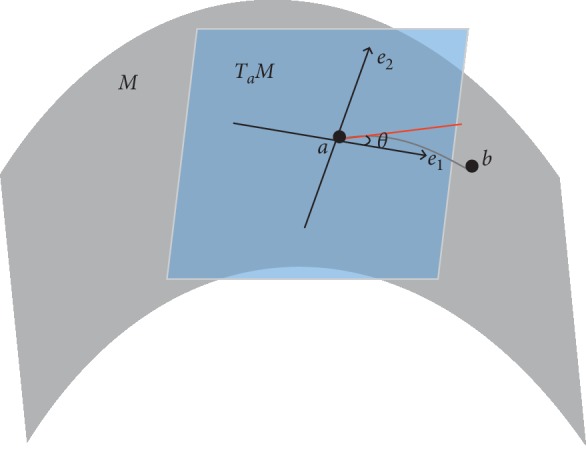
The metric tensor on the surface.

The metric tensor *g*_*a*_ is regarded as a local orthogonal coordinate system in the tangent plane *T*_*a*_*M* (Figure 3), where *e*_1_ and *e*_2_ are the eigenvectors of *g*_*a*_, *λ*_1_ and *λ*_2_ are the eigenvalues of *g*_*a*_, and *θ* is the angle formed between *x* and *e*_1_, then formula (1) can be rewritten as follows:

(2) $$\begin{array}{c} d_{g_{a}}\left(a,b\right)=\left\|x\right\|\sqrt{\lambda_{1}\cos\;{\left(\theta \right)}^{2}+\lambda_{2}\sin\;{\left(\theta \right)}^{2}}. \end{array}$$

If the eigenvectors are fixed, the length is determined by *λ*_1_ and *λ*_2_. Therefore, controlling the value of *λ*_1_ and *λ*_2_ can diminish the distance between two points *a* and *b* in the feature area. Previous researches [1, 31] suggested that the eigenvectors should be aligned with the local curvature directions (minimum curvature and maximum curvature).

**Figure 4:**
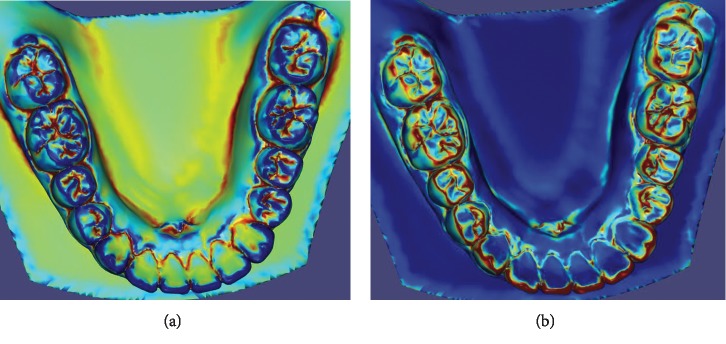
The visual result of (a) |*k*_max_| − |*k*_min_| and (b) |*k*_max_|+|*k*_min_|.

**Figure 5:**
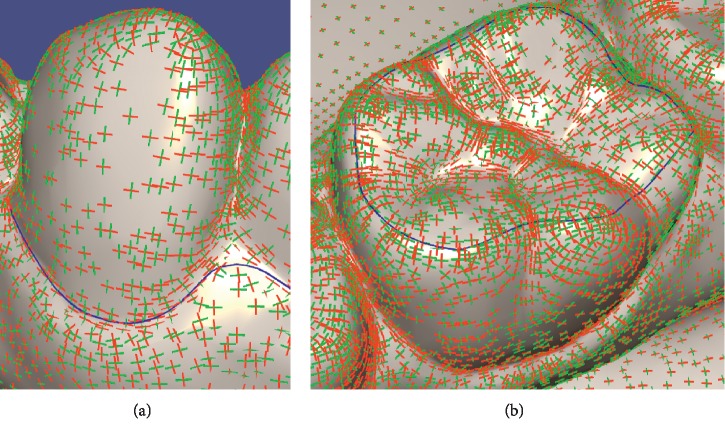
The red line is the direction of minimum curvature, and the green line is the direction of maximum curvature. The blue wires in (a) and (b) are the wires we need.

In this paper, *e*_1_ is aligned with the direction of minimum curvature and *e*_2_ is aligned with the direction of maximum curvature. *k*_min_ and *k*_max_ denote the minimum and maximum curvature at a point of the triangle mesh. |*k*_max_| − |*k*_min_| is smaller in ridges and bigger in valleys, and |*k*_max_|+|*k*_min_| is smaller in valleys and planar area (Figure 4). The tangent direction of wire along valleys is similar to the direction of the minimum curvature (Figure 5(a)). So for valleys, *λ*_1_ and *λ*_2_ are set in formula (3), which is named as valley metric. Dividing by *k*_max_ makes *λ*_1_ and *λ*_2_ scale-independent. It is obvious *λ*_1_ (Figure 4(b)) and sin(*θ*) (Figure 5(a)) are small in valleys, and according to formula (2), the above eigenvalues make the wire along the valleys shorter than that along others:

(3) $$\begin{array}{c} \lambda_{1}=\frac{\left|k_{\max}\right|+\left|k_{\min}\right|}{2\left|k_{\max}\right|},\\ \lambda_{2}=\frac{\left|k_{\max}\right|-\left|k_{\min}\right|}{2\left|k_{\max}\right|}. \end{array}$$

For ridges, *λ*_1_ and *λ*_2_ are set in formula (4), which is named as ridge metric. In ridges, *k*_max_ is similar to *k*_min_ (Figure 4(a)), which means *λ*_2_ is close to 1. Furthermore, in other regions, *λ*_2_ is much more than 1. Besides, because the direction of wire along ridges is similar to the direction of maximum curvature (Figure 5(b)), the smaller *λ*_2_ makes the wire along the ridges shorter. *ε* (10^−4^ in the paper) prevents division by zero. The definitions of *λ*_1_ and *λ*_2_ reveal their scale-independency:

(4) $$\begin{array}{c} \lambda_{1}=1,\\ \lambda_{2}=\frac{\left|k_{\max}\right|}{\left|k_{\min}\right|+\varepsilon }. \end{array}$$

### 3.2. Anisotropic Dijkstra

In order to fast compute the geodesic from all vertices to the source, an anisotropic Dijkstra algorithm is proposed. Its pseudocode is shown in Algorithm 1.

The data structure of the triangle mesh of the teeth as one input of the algorithm is half-edge [32]. Every vertex of the triangle mesh has a set of triples expressed as (dist, final, and pred), where dist is the distance between the vertex and the source, and final marks whether the vertex is visited and pred represents the previous vertex of the vertex in a path.

In the initialisation, dist of source point is zero and all other vertices are infinity. A priority queue *Q* is employed, which is ordered by dist. In the beginning, there is only source vertex in *Q*. Then, until *Q* is empty and in the process dist and pred of all vertices are updated, the main loop is running. In the main loop, the distance between two adjacent vertices-based anisotropic metric is calculated. The metric tensors of the two vertices *v* and *w* are *g*_*v*_ and *g*_*w*_, and distance between *v* and *w* is computed as follows:

(5) $$\begin{array}{c} l_{g}\left(v,w\right)=\frac{d_{g_{v}}\left(v,w\right)+d_{g_{w}}\left(w,v\right)}{2}. \end{array}$$

**Figure 6:**
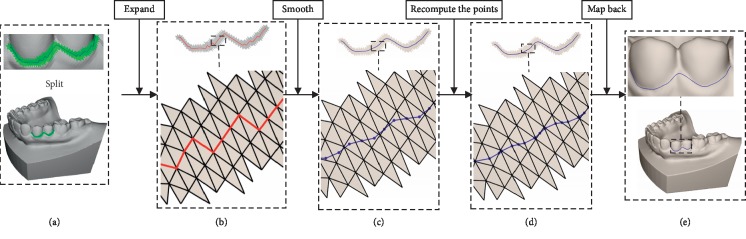
The steps to smooth the 3D curve on the dental mesh.

The geodesic between any vertices and the source vertex is tracked back by pred as shown in Figure 6(a).

### 3.3. Smooth 3D Curve on Dental Mesh

As shown in Figure 6(a), the wire we trace back is jagged, because it consists of the edges of the triangle mesh determined by the Dijkstra algorithm. However, for teeth segmentation, the geodesic should be smooth as the boundary is smooth. To achieve this goal, Polthier and Schmies [33] proposed the “Geodesic Euler Method” and “Geodesic Runge–Kutta Method” based on the gradient of the geodesic field. The methods regard the geodesic tracking back problem as an ordinary differential equation (ODE) solving problem with the initial value, which requires the geodesic field is smooth. However, the geodesic field computed by “Anisotropic Dijkstra” does not satisfy the smooth condition because of its strong anisotropy and weak accuracy. Therefore, Polthier's methods cannot get the correct geodesic. Inspired by common sense that replacing the two endpoints with the midpoint makes polyline smooth on the plane, a 3D midpoint algorithm to smooth curvature on triangle mesh is proposed and the pseudocode is shown in Algorithm 2.

“Midpoint method” is not suitable for smoothing the curve on the 3D triangle mesh because the midpoint of two points in the curve may not locate on the triangle mesh in 3D space. Therefore, in the proposed algorithm, *k* (usually 3 to 5) neighbourhoods of the curve *C* on *triangle_mesh* are extracted (Figure 6(a)), named *k*_*ring_mesh*. Using the method of least squares conformal maps (LSCM) [34], the expanded triangle mesh on the plan is named by *k*_*ring_mesh*_2D (Figure 6(b)). *C* is constructed by the vertices of *triangle_mesh*. The curve *C* is mapped to a 2D curve *C*^2D^ that is constructed by the vertices of *k*_*ring_mesh*_2D. *C*^2D^ is smoothed with the “midpoint method” to *S_C*^2D^ (Figure 6(c)). *S_C*^2D^ is mapped back to a smooth curve *S_C* (Figures 6(d) and 6(e)). The method mainly includes the following steps.

Firstly, the interactions of each edge of *k*_*ring_mesh*_2D and *S_C*^2D^ are computed.

Secondly, one edge of *k*_*ring_mesh*_2D intersects *S_C*^2D^ at *pt_int*^2D^. Two vertices of this edge are defined as index_1_ and index_2_, respectively. Then, the information of intersection is recorded in a set of triples (index_1_, index_1_, and *α*), where *α* is calculated by the following formula:

(6) $$\begin{array}{c} \text{pt}\_{\mathrm{int}}^{2\text{D}}=\left(1-\alpha \right)\cdot {\text{pt}}^{2\text{D}}\left({\text{index}}_{1}\right)+\alpha \cdot {\text{pt}}^{2\text{D}}\left({\text{index}}_{2}\right). \end{array}$$

Finally, *S_C*^2D^ is mapped back to *S_C* by the following formula:

(7) $$\begin{array}{c} \text{pt}\_{\mathrm{int}}^{3\text{D}}=\left(1-\alpha \right)\cdot {\text{pt}}^{3\text{D}}\left({\text{index}}_{1}\right)+\alpha \cdot {\text{pt}}^{3\text{D}}\left({\text{index}}_{2}\right). \end{array}$$

Additionally, pt^2D^ and pt^3D^ are the vertices of *k*_*ring_mesh*_2D and *k*_*ring_mesh*_3D, respectively. And, all the *pt_int*^3D^ form *S_C*. *s* is a parameter of the algorithm to control the smooth strength.

### 3.4. User Interface

Our teeth segmentation tool is developed in C++, depending on libigl [35] and OpenMesh [32]. OpenMesh and libigl mainly provide functions such as half-edge data structure, reading and writing the file, rendering, UI components, and computing some discrete geometry quantities. To begin with, the user holds the Ctrl key and presses the left button of the mouse on the triangle mesh of the teeth model and then a seed is selected. When the Ctrl key is held and the mouse is moving on the triangle mesh, the “shortest” path between the seed and current position of the mouse is acquired and rendered in real time. If the path is realised as “good,” it is confirmed with the Ctrl key and the left mouse button click. When a previous path is regarded as not “good,” it is cancelled by the right mouse button click with holding the Ctrl key, and the previous seed becomes the current one. It is ended by the right button click with Ctrl + Shift holding. Our program stores all the seeds. When the mouse hovers over the seed and the seed is highlighted, then the seed can be dragged to modify the path. During this process, the path is rendered in real time. When the Ctrl key is not held, the mouse is responsible for common 3D intersections including translating, rotating, and scaling.

All the tasks of teeth segmentation, including segmentation between teeth and gums, teeth and teeth, the occlusal surface of the teeth, need the path along valleys or ridges. A radio button is employed to switch the mode (along valleys or ridges). A radio button is to change the metric (formula (3) and (4)) defined in Section 3.1.

## 4. Result and Discussion

### 4.1. Setting

We conducted experiments on clinical dental meshes which are acquired by traditional impression and then 3D scanning using AutoScan-DS100+. All the programs are implemented in C++ and compiled in Microsoft Visual Studio 2017 and run on a computer with 8 GB RAM, Intel® Core™ i7-4790 3.6 GHz CPU, and Windows 10 64 bits system. Real-time interactivity and easy-to-use are significant for the segmentation tool, so they are evaluated in our experiments by comparing with Zhuang's method. In order to evaluate our method, seven dental meshes are used and their information is listed in Table 1.

### 4.2. Results and Analysis

**Figure 7:**
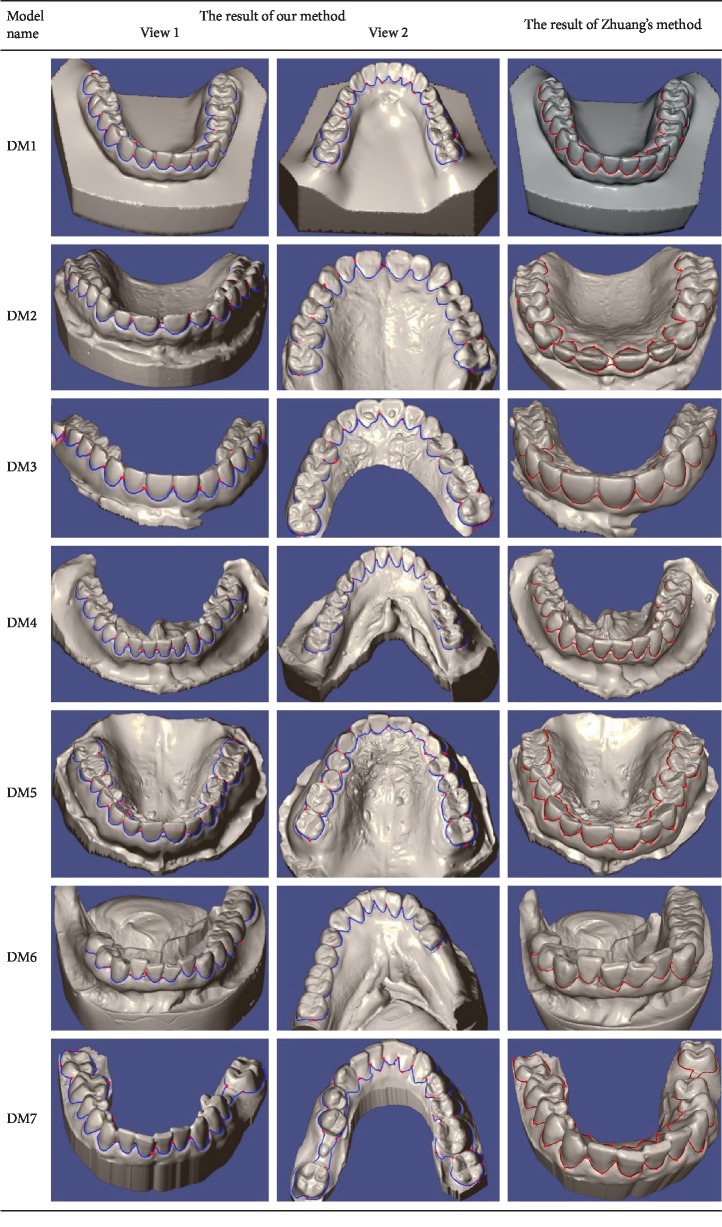
The segmentation boundaries obtained by our method with valley metric and Zhuang's method with his Max metric.

The results of our method are shown in Figure 7. In this experiment, the valley metric is used to segment the teeth and gums from dental mesh with parameters *k* = 3 and *s* = 5. The results of Zhuang's method with his Max metric are given in Figure 7. Furthermore, the parameter settings follow the author's suggestion. Both methods contain three operations: initialisation, seedling, and tracking back path. The running time for every operation is listed in Table 2. In the experiment, the principle of seeds selection is that the wire between two seeds with the furthest distance is as close as possible to the valleys.

As shown in Figure 7, the qualities of the segmentation boundaries acquired by our and Zhuang's method are similar. However, the number of seeds acquired by our method is less than that of Zhuang's method (shown in Table 3). It is evident that, in most cases, the seeding time is much less than one second. The tracking back time is slightly longer than that of Zhuang's method, but a few milliseconds of tracking time does not affect the real-time interaction. So our tool is a WYSIWYG (What You See Is What You Get) program, and the user interface has a pleasant experience. Moreover, the initialisation time of our method is less than that of Zhuang's method. This is because that local subdivision is not required and mesh normalization does not execute with the benefit from our scale-independent metric.

**Figure 8:**
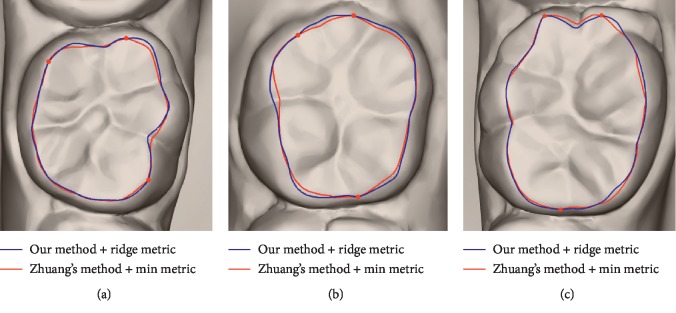
Occlusal surface segmentation of using our method + ridge metric and Zhuang's method + Min metric. The dark blue wire is obtained by our method + ridge metric, and the red one is obtained by Zhuang's method + Min metric.

The comparison between our wire and Zhuang's wire is demonstrated in Figure 1. The blue wire is obtained by valley metric. The red and the dark red wires are obtained by Zhuang's method using his Min and Max metric, respectively. The red points are the seeds, and there are only two seeds at the ends of the wire. The blue and dark red wires overlap each other in Figure 1(a). The wire obtained by the valley metric is closer to the real parting of teeth and gums, especially when the two seeds are far apart, as shown in Figures 1(b)–1(d). It means that the valley metric is more effective than Zhuang's method. Although the performance of ridge metric is close to Zhuang's Min metric shown in Figure 8, our method is faster than Zhuang's method.

**Figure 9:**
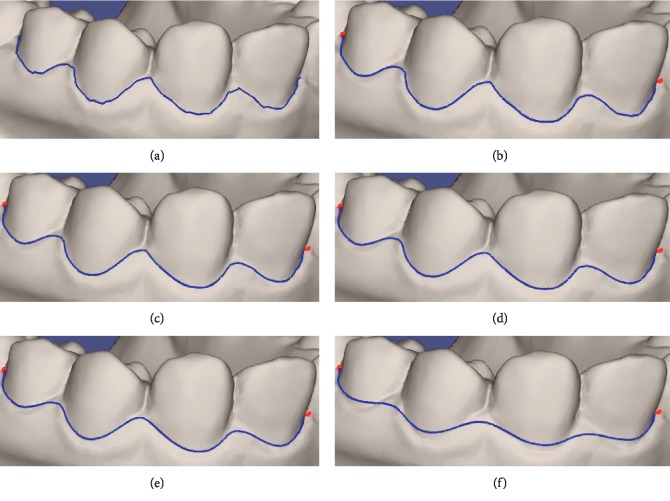
The wires using different smoothing strengths. (a) The original wire, which is not smooth. (b) *s* = 2. (c) *s* = 5. (d) *s* = 10. (e) *s* = 20. (f) *s* = 80.

There are two parameters in the proposed method for smoothing 3D curve. The parameter *k* controls the magnitude of *k*_*ring_mesh*, which is usually set between 3 and 5. A smaller value of *k* may cause that the smoothing curve goes beyond the boundary of the mesh. Moreover, a larger value of *k* inevitably leads to reduction in efficiency because intersections between the curve and the mesh need to be computed. In our tests, when *k* is set to 3, the algorithm performs better. The parameter *s* is the smoothing strength, which affects the times of the taken midpoints. On the plane, the polyline will become a straight line by taking the midpoint enough times. It is also suitable for our 3D curve smoothing method on a triangle mesh. An appropriate value of *s* makes the wire smooth and not far from the original polyline (Figure 9). In our tests, the parameter performs well from 2 to 10. Experimental results show that our method is insensitive to parameters.

**Figure 10:**
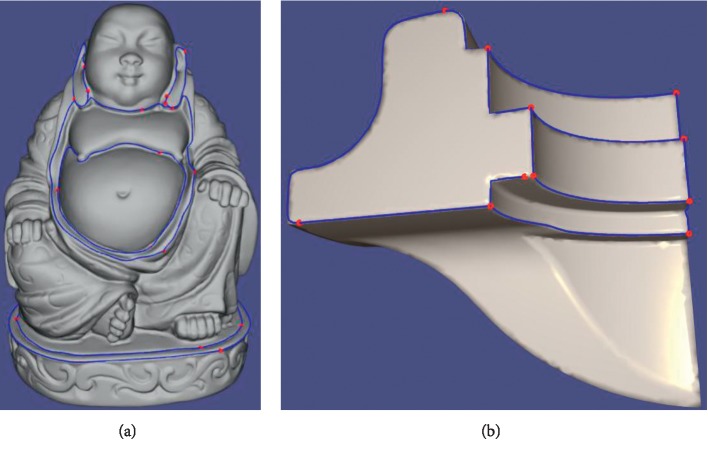
The blue wire is obtained by our tool, and the red points are the seeds.

Our method is proposed for dental mesh segmentation. Meanwhile, it can work well on other triangle meshes with visible valleys or ridges as shown in Figure 10.

## 5. Conclusion

An intelligent scissors tool for teeth segmentation in the dental mesh is proposed, which is inspired by Zhuang's live-wire method. Valley metric and ridge metric are defined to lead the wires along the valleys and the ridges. To quickly compute the geodesic, we adapt the Dijkstra algorithm for the anisotropic metric. Therefore, in order to solve the problem that the path tracked back by Dijkstra is unsmooth, a 3D midpoint smoothing algorithm is proposed. The experiments show that the tool is effective for tasks of teeth segmentation in the dental mesh. Compared to Zhuang's method, our method is better in time complexity and interactivity.

However, the tool is poor in the segmentation of the mesh without prominent valleys or ridges. In future work, it is attractive to design a more versatile metric and to extend the tool for interactive texture mapping.

## Figures and Tables

**Algorithm 1:**
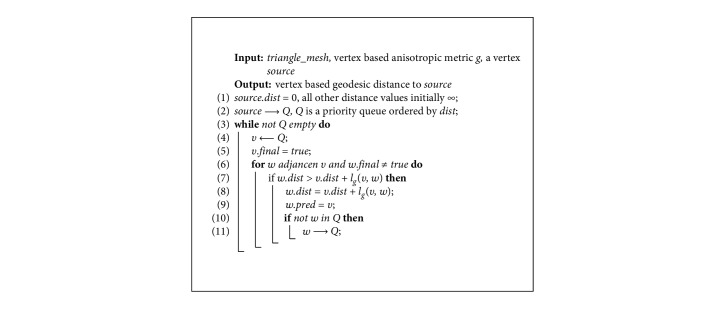
Anisotropic Dijkstra.

**Algorithm 2:**
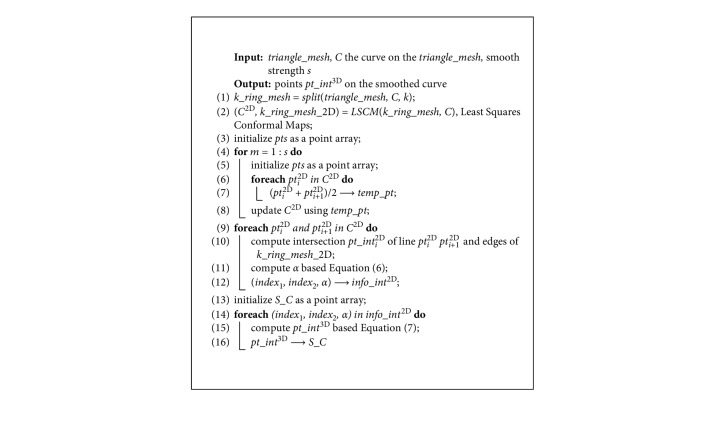
3D midpoint smoothing algorithm.

**Table 1:** The information on the dental meshes.

Name	Number of vertices	Number of faces	Number of edges	Position
DM1	35473	70942	106413	Mandibular
DM2	113489	226843	340325	Maxillar
DM3	84091	168194	252291	Maxillar
DM4	140251	280498	420747	Mandibular
DM5	156257	312510	468765	Maxillar
DM6	226232	452460	678690	Mandibular
DM7	62301	124598	186897	Mandibular

**Table 2:** Running time of every step of our method and Zhuang's method (ms).

Model name	Our method				Zhuang's method			
	Initialisation time	Seeding time	Trackback time	Total time	Initialisation time	Seeding time	Trackback time	Total time
DM1	1206	28	6	2260	1909	654	0.1	35268.1
DM2	3647	92	6	6881	4071	4334	0.2	207778.4
DM3	2707	70	6	5367	3728	2190	0.2	106667.4
DM4	4809	122	8	8449	6108	5432	0.2	266853.6
DM5	5624	140	7	10034	6858	8851	0.2	511376.4
DM6	8505	203	8	13991	9999	10225	0.2	408781.8
DM7	2049	57	5	4281	2306	1208	0.1	60294.8

**Table 3:** The number of seeds used by our method and Zhuang's method.

	DM1	DM2	DM3	DM4	DM5	DM6	DM7
Number of seeds used by our method	31	33	35	28	30	26	36
Number of seeds used by Zhuang's method	51	47	47	48	57	39	48

## Data Availability

The data used to support the findings of this study are available from the corresponding author upon request.

## References

[1] Zhuang Y., Zou M., Carr N., Ju T. (2014). Anisotropic geodesics for live-wire mesh segmentation. *Computer Graphics Forum*.

[2] Mortensen E. N., Barrett W. A. Intelligent scissors for image composition.

[3] Yuan T., Liao W., Dai N., Cheng X., Yu Q. (2010). Single-tooth modeling for 3D dental model. *International Journal of Biomedical Imaging*.

[4] Kronfeld T., Brunner D., Brunnett G. (2010). Snake-based segmentation of teeth from virtual dental casts. *Computer-Aided Design and Applications*.

[5] Wu K., Chen L., Li J., Zhou Y. (2014). Tooth segmentation on dental meshes using morphologic skeleton. *Computers & Graphics*.

[6] Liao S.-h., Liu S.-j., Zou B.-j., Ding X., Liang Y., Huang J.-h. (2015). Automatic tooth segmentation of dental mesh based on harmonic fields. *BioMed Research International*.

[7] Li Z., Wang H. (2016). Interactive tooth separation from dental model using segmentation field. *PLoS One*.

[8] Chen X., Golovinskiy A., Funkhouser T. (2009). A benchmark for 3D mesh segmentation. *ACM Transactions on Graphics*.

[9] Rodrigues R. S. V., Morgado J. F. M., Gomes A. J. P. (2018). Part-based mesh segmentation: a survey. *Computer Graphics Forum*.

[10] Chazelle B. (1997). Strategies for polyhedral surface decomposition: an experimental study. *Computational Geometry*.

[11] Bergamasco F., Albarelli A., Torsello A. Semi-supervised segmentation of 3D surfaces using a weighted graph representation.

[12] Bergamasco F., Albarelli A., Torsello A. (2012). A graph-based technique for semi-supervised segmentation of 3D surfaces. *Pattern Recognition Letters*.

[13] Benjamin W., Polk A. W., Vishwanathan S. V. N., Ramani K. (2011). Heat walk: robust salient segmentation of non-rigid shapes. *Computer Graphics Forum*.

[14] Shlafman S., Tal A., Katz S. (2010). Metamorphosis of polyhedral surfaces using decomposition. *Computer Graphics Forum*.

[15] Saha R., Donofrio R. S., Goeres D. M., Bagley S. T. (2012). Variational mesh decomposition. *ACM Transactions on Graphics*.

[16] Katz S., Tal A. (2003). Hierarchical mesh decomposition using fuzzy clustering and cuts. *ACM Transactions on Graphics*.

[17] Zhang H., Li C., Gao L., Li S., Wang G. (2015). Shape segmentation by hierarchical splat clustering. *Computers & Graphics*.

[18] Golovinskiy A., Funkhouser T. (2008). Randomized cuts for 3D mesh analysis. *ACM Transactions on Graphics*.

[19] Zheng Y., Tai C.-L. (2010). Mesh decomposition with cross-boundary brushes. *Computer Graphics Forum*.

[20] Zheng Y., Tai C.-L., Au O. K.-C. (2012). Dot scissor: a single-click interface for mesh segmentation. *IEEE Transactions on Visualization and Computer Graphics*.

[21] Zhao J., Xin S., Liu Y. (2015). A survey on the computing of geodesic distances on meshes. *Scientia Sinica Informationis*.

[22] Chen J., Han Y. Shortest paths on a polyhedron.

[23] Surazhsky V., Surazhsky T., Kirsanov D., Gortler S. J., Hoppe H. (2005). Fast exact and approximate geodesics on meshes. *ACM Transactions on Graphics*.

[24] Ying X., Wang X., He Y. (2013). Saddle vertex graph (SVG). *ACM Transactions on Graphics*.

[25] Campen M., Heistermann M., Kobbelt L. (2013). Practical anisotropic geodesy. *Computer Graphics Forum*.

[26] Crane K., Weischedel C., Wardetzky M. (2013). Geodesics in heat. *ACM Transactions on Graphics*.

[27] Crane K., Weischedel C., Wardetzky M. (2017). The heat method for distance computation. *Communications of the ACM*.

[28] Yang F., Cohen L. D. (2016). Geodesic distance and curves through isotropic and anisotropic heat equations on images and surfaces. *Journal of Mathematical Imaging and Vision*.

[29] Andreux M., Rodolà E., Aubry M., Cremers D. Anisotropic Laplace-Beltrami operators for shape analysis.

[30] Dijkstra E. W. (1959). A note on two problems in connexion with graphs. *Numerische Mathematik*.

[31] Vaxman A., Campen M., Diamanti O. (2016). Directional field synthesis, design, and processing. *Computer Graphics Forum*.

[32] Botsch M., Steinberg S., Bischoff S., Kobbelt L., Aachen R. OpenMesh: a generic and efficient polygon mesh data structure.

[33] Polthier K., Schmies M. Straightest geodesics on polyhedral surfaces.

[34] Lévy B., Petitjean S., Ray N., Maillot J. (2002). Least squares conformal maps for automatic texture atlas generation. *ACM Transactions on Graphics*.

[35] Jacobson A., Panozzo D., Schüller C. https://libigl.github.io/.

